# A selected reaction monitoring mass spectrometric assessment of biomarker candidates diagnosing large-cell neuroendocrine lung carcinoma by the scaling method using endogenous references

**DOI:** 10.1371/journal.pone.0176219

**Published:** 2017-04-27

**Authors:** Tetsuya Fukuda, Masaharu Nomura, Yasufumi Kato, Hiromasa Tojo, Kiyonaga Fujii, Toshitaka Nagao, Yasuhiko Bando, Thomas E. Fehniger, György Marko-Varga, Haruhiko Nakamura, Harubumi Kato, Toshihide Nishimura

**Affiliations:** 1 Biosys Technologies, Inc., Tokyo, Japan; 2 Department of Thoracic and Thyroid Surgery, Tokyo Medical University, Tokyo, Japan; 3 Department of Thoracic Surgery, Tokyo Medical University Ibaraki Medical Center, Ibaraki, Japan; 4 Department of Biophysics and Biochemistry, Osaka University Graduate School of Medicine, Osaka, Japan; 5 Department of Translational Medicine Informatics, St. Mariana University School of Medicine, Kawasaki, Japan; 6 Department of Clinical Pathology, Tokyo Medical University, Tokyo, Japan; 7 Center of Excellence in Biological and Medical Mass Spectrometry, Lund University, Lund, Sweden; 8 Clinical Protein Science & Imaging, Biomedical Center, Department of Biomedical Engineering, Lund University, Lund, Sweden; 9 Department of Chest Surgery, St. Marianna University School of Medicine, Kawasaki, Japan; 10 Chest Surgery, Niizashiki Central General Hospital, Saitama, Japan; H Lee Moffitt Cancer Center and Research Institute, UNITED STATES

## Abstract

Selected reaction monitoring mass spectrometry (SRM-MS) -based semi-quantitation was performed to assess the validity of 46 selected candidate proteins for specifically diagnosing large-cell neuroendocrine lung carcinoma (LCNEC) and differentiating it from other lung cancer subtypes. The scaling method was applied in this study using specific SRM peak areas (AUCs) derived from the endogenous reference protein that normalizes all SRM AUCs obtained for the candidate proteins. In a screening verification study, we found that seven out of the 46 candidate proteins were statistically significant for the LCNEC phenotype, including 4F2hc cell surface antigen heavy chain (4F2hc/CD98) (*p*-ANOVA ≤ 0.0012), retinal dehydrogenase 1 (*p*-ANOVA ≤ 0.0029), apolipoprotein A-I (*p*-ANOVA ≤ 0.0004), β-enolase (*p*-ANOVA ≤ 0.0043), creatine kinase B-type (*p*-ANOVA ≤ 0.0070), and galectin-3-binding protein (*p*-ANOVA = 0.0080), and phosphatidylethanolamine-binding protein 1 (*p*-ANOVA ≤ 0.0012). In addition, we also identified candidate proteins specific to the small-cell lung carcinoma (SCLC) subtype. These candidates include brain acid soluble protein 1 (*p*-ANOVA < 0.0001) and γ-enolase (*p*-ANOVA ≤ 0.0013). This new relative quantitation-based approach utilizing the scaling method can be applied to assess hundreds of protein candidates obtained from discovery proteomic studies as a first step of the verification phase in biomarker development processes.

## Introduction

The challenge for healthcare is to provide the right medication at the right time to the right patient at a minimal cost. Thus, diagnosing the right disease subtype for each patient is the most important step to circumvent complicated and lengthy medical treatments [1]. Accurate and specific biomarkers for each disease subtype are expected to provide a targeted treatment by personalized medicine [2]. Biomarker development toward clinical application undergoes the following three phases: i) discovery, ii) verification, and iii) finally validation [3]. The discovery of biomarker candidates can be conducted by a comparative proteomic study utilizing clinical specimens, with well-designed protocols in a collaborative effort between clinicians and scientists.

Most exploratory clinical proteomic studies make use of a label-free discovery proteomic analysis platform, which is based on high-resolution liquid chromatography separation, interfaced with tandem mass spectrometry. This analysis procedure has proven powerful in biomarker discovery studies, utilizing clinical samples to screen large numbers of proteins in a low number of samples, and had led to the identification of 100 or more biomarker candidates being differentially expressed [4–6].

Within oncology, lung cancer is the most common global cancer disease, and the major cause of death [7–8]. Large-cell lung cancer (LCC) is a subtype of cancerous lung cells, in which cells proliferate and grow without any distinctive tissue construct. Small-cell lung cancer (SCLC), a pulmonary neuroendocrine tumor (NET), is the most common lung cancer. SCLC accounts for an estimated 28,000 of the 219,440 lung cancer cases diagnosed in the United States in 2009 [7]. Travis et al. [9] were the first to propose a new subtype of LCC, named large-cell neuroendocrine lung carcinoma (LCNEC). LCNEC is often generically grouped among non-small cell lung cancers (NSCLCs) [10]. The LCNEC group is a new pathological phenotype; LCNEC are highly malignant neoplasms arising from transformed epithelial cells originating in tissues) within the pulmonary tree. SCLC is another lung cancer phenotype, recognized as an aggressive NET consisting of small bare nuclei cells. Both LCNEC and SCLC belong to NET of the lung according to the WHO classification (2015) [11]. To the best of our knowledge, there are still no approved LCNEC biomarkers; thus, treatment strategies with LCNEC biomarker diagnosis would benefit patients, who could then receive a tailored treatment [12].

In a previous study, we performed laser capture microdissection (LCM) of cancerous cells from archived FFPE tissue specimens and analyzed lung cancer subtypes by mass spectrometry (MS) -based discovery sequencing [13]. We had identified several hundreds of proteins differentially expressed, which would be correlated with LCNEC. Among those candidates, we examined four proteins: retinal dehydrogenase 1 (AL1A1), aldo-keto reductase family 1 members C1 (AK1C1) and C3 (AK1C3), and CD44 antigen by immunohistochemistry [13].

LCM of tumor cells results in very small amounts of proteins, insufficient for conventional protein assays. The use of Absolute QUAntitation (AQUA) peptides/proteins allows obtaining absolute quantitation for limited number of targeted peptides/proteins [14,15]. However, this challenging experiment might not necessarily answer important biological questions, in particular when related to the functional role of targeted proteins and protein complexes.

Liquid chromatography (LC) / selected- or multiple- reaction monitoring (SRM or MRM) MS of peptides using stable isotope dilution (SID) has been proven to be powerful for targeted protein quantitation [14, 16, 17]. However, according to Liebler et al. [18], the high cost of labeled peptide standards for SID makes it realistically difficult to conduct SRM studies. SID-LC/SRM-MS measurements for three specific peptides each designed for 50 proteins would cost about $150,000. They compared SID to a labeled reference peptide method, which uses a single labeled peptide as a reference standard for all measured peptides, and a label-free approach, in which quantitation is based on analysis of un-normalized peak areas for detected MRM transitions. Liebler et al. [18] concluded that both labeled reference peptide and label-free methods are cost-effective alternative to SID for many runs of LC/SRM-MS measurements. The expense of labeled standard peptides is indeed the big barrier to conduct “verification” for hundreds of protein biomarker candidates, resulting from discovery proteomic studies. Therefore, a cost-effective screening of biomarker candidates is recommended to narrow down the numbers into promising candidates. Then, highly accurate quantitation assays based on SRM-MS utilizing, as less as possible, isotopically labeled peptide standards are applied for further verification.

Relative quantitation of candidate proteins from SRM-MS based assessment can be obtained by the scaling method utilizing the endogenous protein reference. Validation by Western blotting [19] is popular for relative quantitation using housekeeping proteins expressed ubiquitously, including β-actin, β-tubulin, and glyceraldehyde phosphate dehydrogenase (GAPDH), as loading controls or protein references.

Applications to clinical proteome studies are very popular [20, 21], where comparative analyses are carried out within various disease cells of different subtypes and stages, in relation to controls and healthy subjects. The multifunctional Parkinson’s disease protein 7/deglycase-1 (PARK7/DJ-1) [22] was proposed as the protein with the lowest variability from expression data across diverse comparisons between tissue and cells originating from evolutionary distant vertebrate species. A recent report provided data on the upregulation of PARK7/DJ-1 and its association with drug-induced apoptosis [23]. Disease areas examined included cisplatin resistance in NSCLC [24], oxidative stress-response in human pneumocytes [25], and cell proliferation, and migration/invasion in lung adenocarcinoma [26], predicting NSCLC [27].

Here we report a SRM-MS quantitation (i.e., targeted proteomics) [8] capable of targeting candidate proteins (as many as qualified to be statistically significant) in the exploratory proteomic analysis. This SRM -MS semi-quantitative scaling method is using the application of endogenous references [28, 29] to assess 46 proteins chosen as the subset of LCNEC biomarker candidates.

## Material and methods

### FFPE tissues and sample preparation

**Fig 1:**
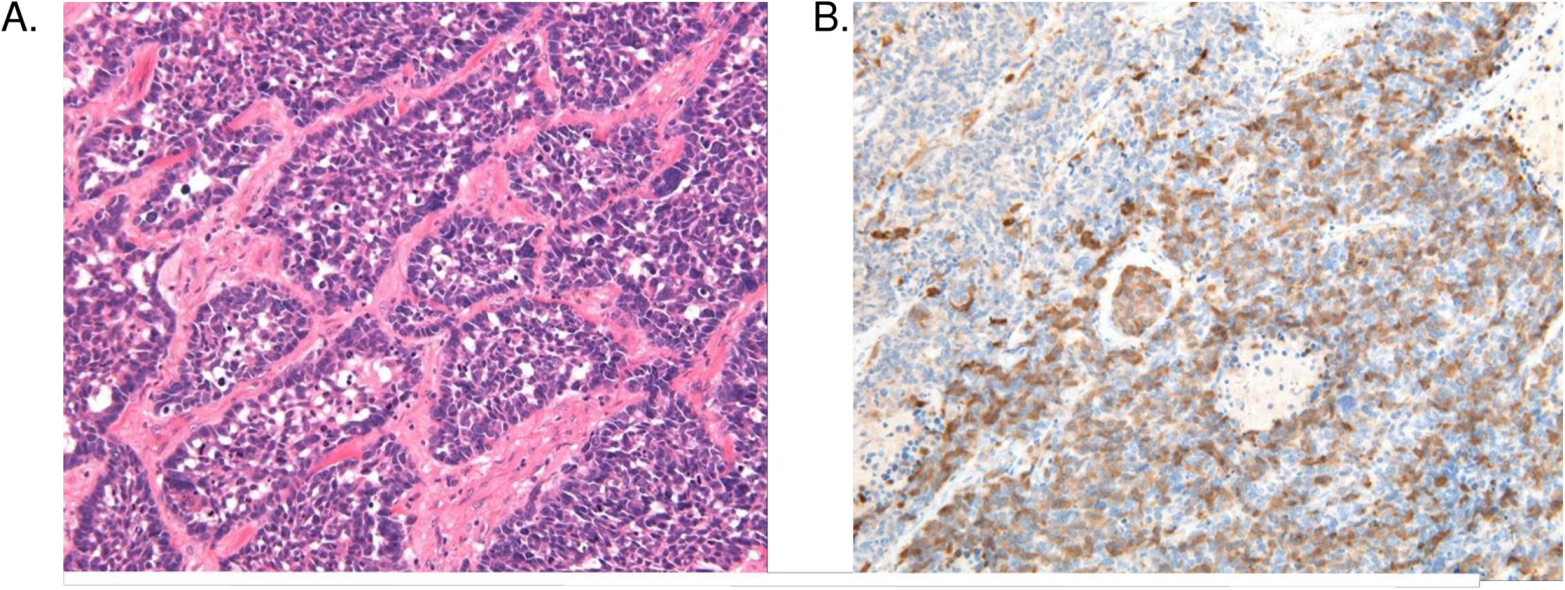
A) Histological appearance of LCNEC (Patient No. 2 of LCNEC in S1 Table, magnified by ×200), and B) Immunohistochemical staining (×200) of AL1A1, using monoclonal rabbit AL1A1 antibody (Abcom Japan, Tokyo, Japan).

The study protocol is compliant with the principles of the declaration of Helsinki. All patients (*n* = 30) signed an informed consent and the study protocol was approved by the ethics committee of the Tokyo Medical University Hospital. Cancerous lesions were identified on serial tissue sections stained with hematoxylin and eosin (HE). Fig 1 shows an example of a histological patient tumor from the LCNEC subtype (Patient No. 2 of LCNEC on S1 Table [13, 30], magnified by ×200). Cancer cells are observed within the tumor tissue, with relatively distinct nucleoli and large cytoplasm, revealing the palisading pattern at the periphery of the nests with rosettes-like formations. For proteomic analysis, a 10 μm thick section was prepared from the same tissue block and attached onto the DIRECTOR™ slides (OncoPlexDx, Rockville, MD, USA). These sections were de-paraffinized twice with xylene for 5 min, rehydrated with graded ethanol solutions and distilled water, and stained by hematoxylin. Those slides were air-dried and subjected to LCM with a Leica LMD6000 (Leica Micro-systems GmbH, Ernst-Leitz-Strasse, Wetzlar, Germany). Laser capture microdissected areas were collected directly into a 200 μL low-binding plastic tube, corresponding to approx., 8.0 mm^2^ and 30,000 cells per tissue. Diagnosis was made using the 4 μm sections stained with HE. Pathologists provided their diagnosis independently according to the WHO classification [30] at the Department of Clinical Pathology, Tokyo Medical University Hospital. LCNEC is a characteristic cancer cell with relatively larger cytoplasm, less fine chromatin, and more distinct nucleoli than SCLC cells. The exemplified sections from patients were diagnosed unequivocally and used in this study.

Proteins were extracted and digested with trypsin using Liquid Tissue™ MS Protein Prep kits (OncoPlexDx) according to the manufacturer's protocol [31].

### Liquid chromatography-SRM tandem mass spectrometry

The capillary reversed-phase μ-LC MS/MS system comprises a Paradigm MS4 dual solvent delivery system (Michrom BioResources, Auburn, CA, USA) interfaced with the AD-H6 closed electrospray ionization (ESI) interface (AMR Inc., Tokyo, Japan) to a hybrid triple quadrupole/linear ion trap mass spectrometer (4000-QTRAP, AB Sciex, Foster City, CA, USA) operating in the positive ion mode [29, 32, 33]. The samples subjected to the SRM-MS assay in this study were prepared from FFPE tissues of LCNEC Patients No. 1–4, LCC Patient No 11–15, and SCLC Patient No. 21–25 listed in S1 Table. A 1.5 μL aliquot of each sample (0.15–0.3 μg total peptide) was desalinated on line with an L-trap micro cartridge (0.3 x 5 mm, 5 μm in diameter; Chemicals Evaluation Research Institute, Tokyo, Japan) fitted to the autosampler HTC-PAL injector valve (CTC Analytics, Zwingen, Switzerland), which had been pre-equilibrated with 0.1% trifluoroacetic acid (aq.) containing 2% acetonitrile. After switching the valve, peptides were loaded on a capillary reversed-phase column (0.1 × 150 mm) packed with L-column Micro C18 (3 μm in diameter, and 12 nm pore size; Chemicals Evaluation and Research Institute, Tokyo, Japan) for separation. Mobile phase A was 98% water/2% acetonitrile /0.1% formic acid, and mobile phase B was 10% water/90% acetonitrile/0.1% formic acid. Peptides were eluted from the column with a linear gradient from 95:5; mobile phase A/mobile phase B to 45:55; mobile phase A/mobile phase B over 60 min. at a flow rate of 500 nL/min. The eluent was transferred into the AD-H6 spray head (AMR Inc., Tokyo, Japan) via coupling to a distal coated PicoTip fused silica spray tip (360 μm o.d., 50 μm i.d., 10 μm emitter orifice; New Objective, Woburn, MA, USA). Samples were analyzed with an ionspray voltage of 1.8 kV, heater interface temperature of 150°C, curtain gas flow of 0 and nebulizing gas flow of 5. For all SRM studies, quadrupoles were operated in the unit/unit resolution, dwell times of 25 ms, and collision energy (CE) was determined using the equation: CE = 0.044 × *m/z* + 6 for doubly-charged precursor ions [29].

### Selection of SRM transitions

**Fig 2:**
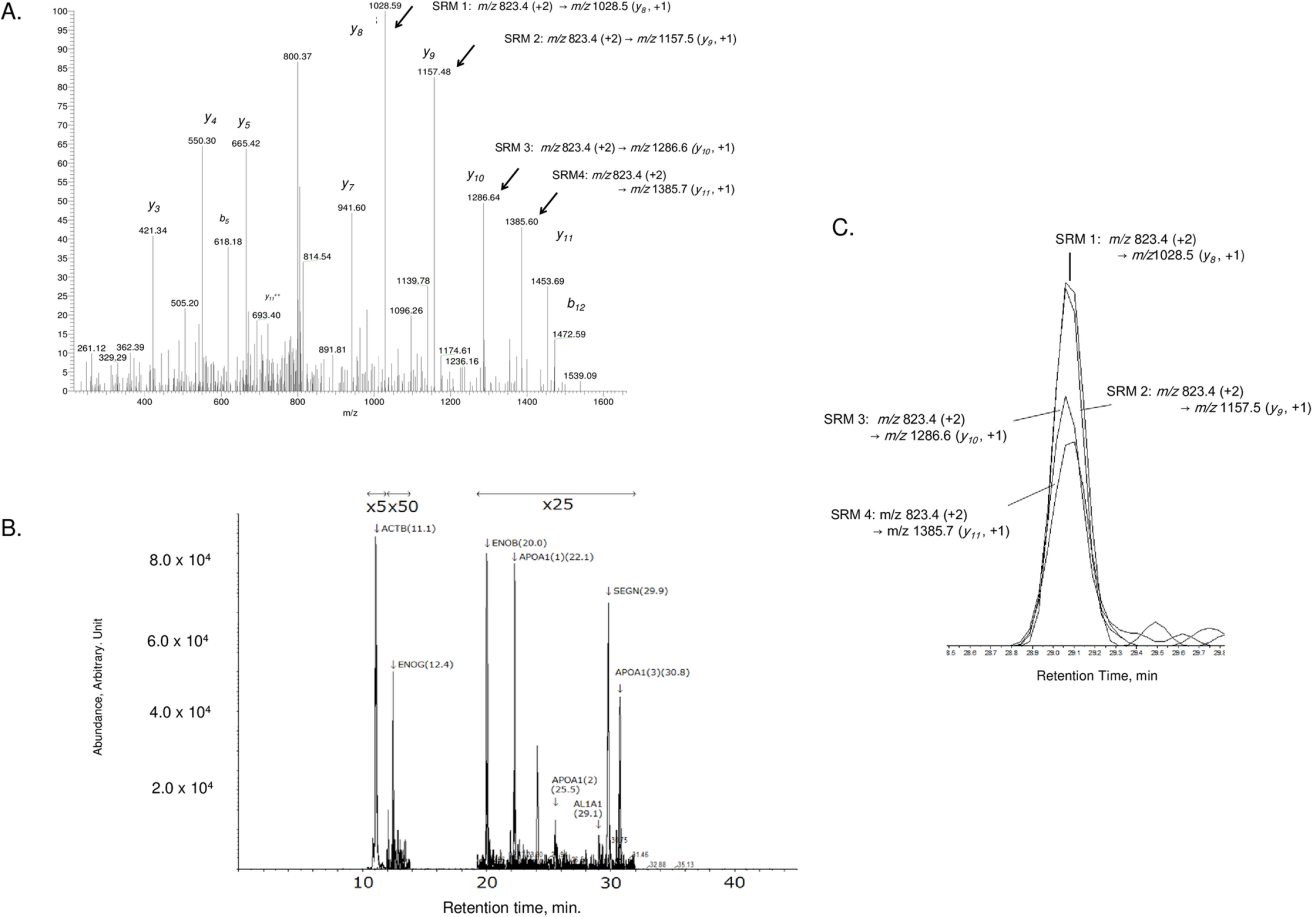
A targeted MS-based quantitation using the SRM transitions specific to a peptide/protein. A) A MS/MS spectrum of the AL1A1 peptide ion, IFVEESIYDEFVR (*m/z* 823.4, 2+), measured on a LTQ-Orbitrap hybrid mass spectrometer, where arrows indicate its four selected fragment ions (*y*_*8*_, *y*_*9*_, *y*_*10*_, and *y*_*11*_) being utilized to construct four SRM transitions. B) An extracted ion chromatogram (XIC) comprised of representative SRM transition peaks measured on a 4000-QTRAP hybrid triple quadrupole/linear ion trap mass spectrometer, where the protein names, their peptide sequences with retention time are denoted: ACTB (AGFAGDDAPR), ENOG (IEEELGDEAR), ENOB (VNQIGSVTESIQACK), APOA1 (1, DYVSQFEGSALGK; 2, LLDNWDSVTSTFSK; 3, VSFLSALEEYTK), AL1A1 (IFVEESIYDEFVR), and SEGN (LDAAGFWQVWQR). C) The four SRM transition peaks for AL1A1.

A total number of 1,981 proteins were identified from the three cancer groups [13], using strict criteria that included statistical evaluation by G-test [34], followed by further elimination of the housekeeping proteins. Biomarker candidates were obtained by spectral counting approaches from pairwise group-comparisons of proteins identified in exploratory proteomic analyses [13]. There, in both LCNEC versus SCLC and LCNEC versus LCC, a protein is significant to LCNEC when its *p*-value in pairwise G-test < 0.05, its |*R*_*SC*_ |≥ 1, and its expression was detected for >50% of total LCNEC samples. Herein, *R*_*SC*_ is the fold change of expressed protein in the base 2 logarithmic scale [35]. Similarly, in both pairwise comparisons, proteins significant to SCLC and LCC were obtained, in which some of proteins significant to SCLC were chosen to be subjected to SRM-MS assays since it is more useful to distinguish between LCNEC and SCLC. Out of more than hundred protein candidates, 46 proteins were selected as the subset of biomarker candidates in this study and were subjected to SRM-MS verification experiments. In total, 69 peptides were targeted along with their corresponding 169 SRM transitions. Resulting data is illustrated in Fig 2A, showing a typical MS/MS spectrum of the AL1A1 doubly-charged peptide, IFVEESIYDEFVR (*m/z* 823.4), in which fragment ions utilized for its SRM transitions are indicated by arrows. In Fig 2C the corresponding four SRM transitions are shown with AUCs measured. In S2 Table, we summarized all the targeted peptides of the candidate biomarker proteins empirically chosen, using data obtained in the exploratory FFPE tissue LC MS/MS experiments [13] along with the SRM transitions constructed with the multiple reaction monitoring (MRM) -initiated detection and sequencing (MIDAS) [29, 32]. The SRM transitions for candidate peptides were evaluated using a 4000-QTRAP instrument since fragmentation profiles being obtained in a LTQ linear ion trap (IT)/Orbitrap mass spectrometer generally differ from those in a triple quadrupole/linear IT mass spectrometer. Selected peptide ions were mostly of 7–16 amino acid length in positive doubly-charged state.

### Scaling method by endogenous protein reference

A comparison of target protein expression levels among several different clinical samples can be obtained so that an experimental SRM AUC of a targeted protein is scaled by the endogenous protein references [13, 29]. The endogenous reference could be selected from well-characterized housekeeping proteins such as GAPDH, β-actin, tubulin, and histones that are used as a general measure of protein expression levels in cells [19, 20]. In particular, β-actin and histone H4 were chosen as endogenous protein references because both are highly expressed in NSCLC cells with relatively low variations in label-free semi-quantitative spectral counting-based comparisons [13, 29].

In this study, the protein reference is chosen among proteins expressed with minimal variations from pairwise comparative analyses in exploratory proteomics (LCNEC versus SCLC and LCNEC versus LCC) under the following criteria: spectral counts (*SpI*) > 30, |*R*_*SC*_| < 0.5, |*NSAF*| > 0.0005, amino acid number (AA) of a protein < 500, and non-significant level in pairwise G-statistics. Herein, *NSAF* is the normalized spectral abundance factor, which is based on spectral counting [36].

### Statistical evaluation of SRM-MS semi-quantitation

Normalized AUCs obtained for individual SRM transitions were subjected to ANalysis Of VAriance (ANOVA) -test, T-test, and both non-parametric Kruskal–Wallis test and non-parametric pairwise Wilcoxon test to calculate their *p*-values (JMP software; SAS Institute, Cary, NC.).

## Results and discussion

To date, the challenge remains to diagnose and treat lung cancer patients in an optimal way. In this respect defining the characteristics of LCNEC (within the lung), some years ago, was an important step to aid in the stratification of patients in disease presentation. We performed a discovery proteomics study with extensive deep mining and disease specific protein expressions as an outcome [13]. The diagnosis of LCNEC requires attention to neuroendocrine features by light microscopy and confirmation by immunohistochemical staining for neuroendocrine markers. Both SCLC and pulmonary LCNEC are high-grade and poor prognosis tumors, with higher incidence in males and smokers [37].

To perform a qualitative screening for LCNEC specific proteins, we included two additional patient groups in the study, the LCC and the SCLC phenotypes, allowing us to evaluate both selectivity and specificity. This is crucial as lung NETs were diagnosed by pathological investigations to be heterogeneous, as seen on the tissue morphology images in Fig 1. LCNEC is a subtype of pulmonary cancers representing approximately 20% of all lung cancers, including SCLC and LCNEC.

In the current study, cancer cells were isolated by LCM from the FFPE tissue specimens as described in the experimental section. Consequently, three aliquots from each sample were analyzed by *nano*-LC/SRM-MS in random sequences. Both the tryptic peptides of bovine serum albumin (BSA /QC control) and the project standard (a mixture of equal volumes of all FFPE samples (*n* = 14) (LCNEC, Patient No. 1–4; LCC, Patient No. 11–15; SCLC, Patient No. 21–25) were measured every 7th run of the FFPE cycle. To ensure true positive peaks, SRM transition peaks of each peptide were examined in its extracted ion chromatogram (XIC) (as shown in Fig 2B). Next, each SRM AUC was extracted with Multi Quant™ Ver. 2.0.2 from SRM XIC data. Peak areas in XIC were normalized by those of the reference as described in the previous section.

We also developed a scaling method by the endogenous protein reference. In our previous study [13] the fold change of expressed protein (*R*_*SC*_) was 0.360 for β-actin and 0.928 for PARK7/DJ-1, comparing LCNEC vs. SCLC, respectively. The housekeeping protein β-actin is highly expressed in NSCLC cells with relatively low variations in label-free semi-quantitative spectral counting analyses among lung cancer subtypes [13, 28, 29]. Still, β-actin could be useful as the endogenous reference in MS-based comparative proteomes. The scaling principle method might be viewed as *MS-based Western blotting*, deprived of the weaknesses accompanying antibody-based quantitation.

Instead, when following determination of a protein reference among proteins identified in comparative proteomic analyses, SRM AUCs measured for targeted candidate proteins/peptides are scaled by the reference, and are evaluated via statistical tests. The scaling method serves as a preliminary screening for over 100 biomarker protein candidates obtained from the discovery proteomic study. It should be, however, noted that this scaling method remains an issue accompanied by its quantitative correctness in comparison. Absolute quantitative SRM-MS assays using isotope-labeled standard peptides are further applied on a smaller number of selected protein candidates.

**Fig 3:**
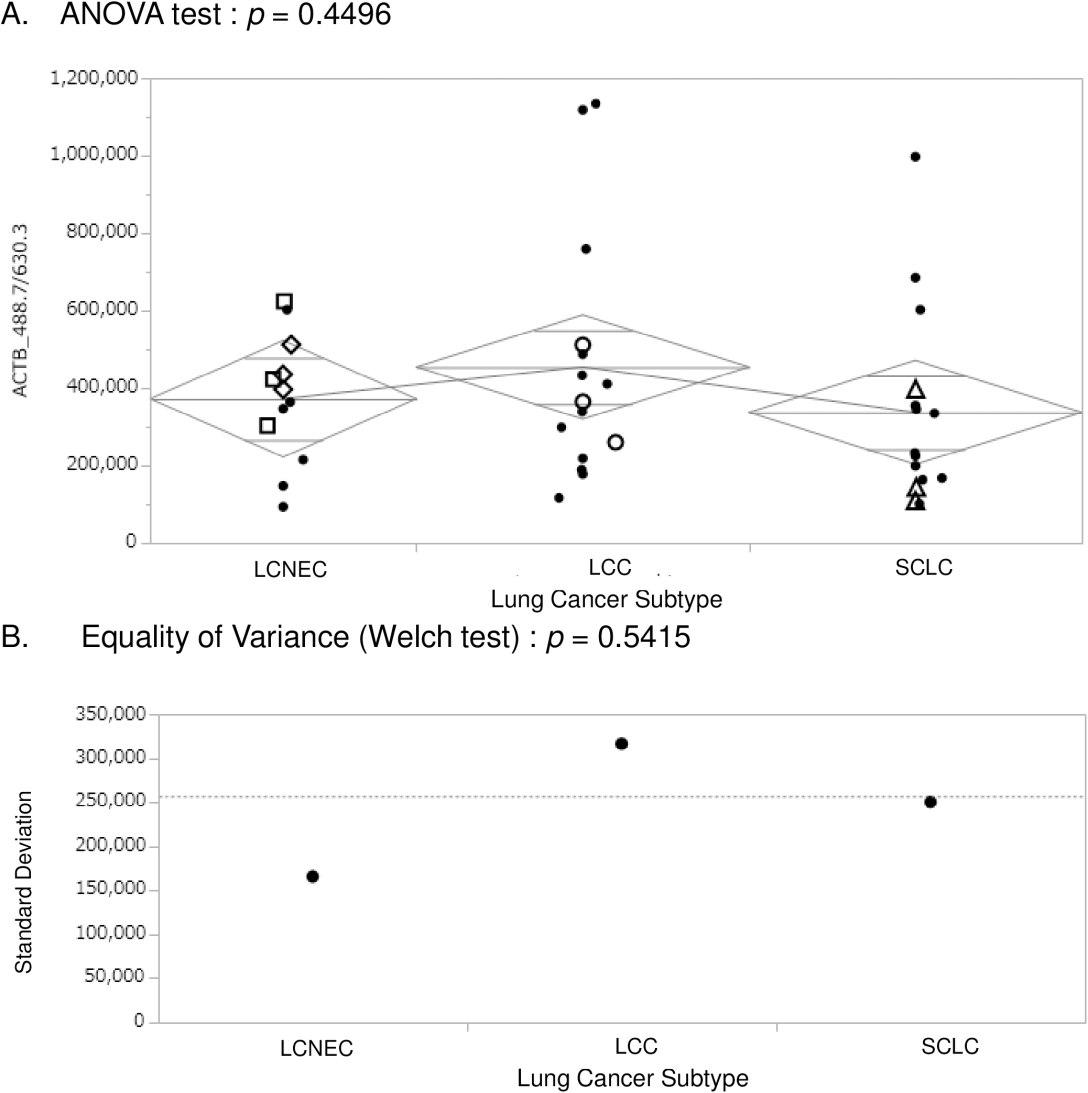
Statistical evaluation for the 42 SRM AUC raw data obtained for the SRM transition of ACTB_488.7/630.3 [doubly-charged β-actin peptide AGFAGDDAPR (*m/z* 488.7) to the singly-charged *y*_*6*_ fragment (*m/z* 630.3)] measured in triplicate across all the 14 clinical samples. The raw SRM AUCs obtained by triplicate LC/SRM-MS runs for some of samples are also indicated (□: LCNEC, Patient No. 1; ◇: LCNEC, Patient No. 4; ○: LCC, Patient No. 13; △: SCLC, Patient No. 24).

Fig 3 shows the statistical evaluation for the 42 raw SRM AUCs obtained for the SRM transition of ACTB_488.7/630.3 [doubly-charged actin-β peptide AGFAGDDAPR (*m/z* 488.7) to the singly-charged *y*_*6*_ fragment (*m/z* 630.3)] measured in triplicate across all the 14 clinical samples. There was no difference among SRM AUCs for each lung cancer group subtypes, LCNEC, LCC, and SCLC [*p* = 0.4496 by ANOVA and *p* = 0.5415 by equality of variance (Welch test)]. We adopted this specific SRM transition as the endogenous reference or normalizer since all experimental AUCs obtained for targeted proteins in different samples were normalized and so that the reference AUC in respective LC/SRM-MS run be 500,000. However, regardless of this reasonably stable expression of β-actin, the scattered SRM AUC values observed can be brought about from unevenness over samples, technical variation in every LC/SRM-MS run, and/or inadequate calculations of SRM AUCs from extracted SRM ion chromatograms. In addition, the actin expression level varies in different parts of tissues or different cell types. A detailed listing of all the respective coefficients of variation, with CV-% values for scaled/normalized AUCs (169 SRM measurements) are provided in S2 Table, using the project standard pooled sample (prepared as a mixture of each aliquot of digested LCNEC, LCC, and SCLC samples) ranging between 4.1 and 115.5%, with an average of 32.9%.

After scaling the data with the reference methodology, we assigned criteria for selecting a protein as a marker candidate for the LCNEC subtype as follows:

1. the protein is from the LCNEC subtype,
2. the relative abundance throughout three subtypes in averaged SRM AUC is >50%, with a *p*-value < 0.01 in ANOVA, obtained by triplicate runs,
3. the analysis includes pairwise *p*-values < 0.01 in the T-test for both LCNEC vs. LCC and LCNEC vs. SCLC.

All the data are compiled and presented in Table 1, summarizing the normalized SRM AUCs of the seven retained candidates. The data was validated as being statistically significant for the LCNEC subtype. The validated candidate proteins were; the cell surface antigen heavy chain (4F2hc), retinal dehydrogenase 1 (AL1A1), apolipoprotein A-1 (APOA1), β-enolase (ENOB), creatine kinase B-type (KCRB), galectin-3-binding protein (LG3BP) and phosphatidylethanolamine-binding protein 1 (PEBP1). The entire data set with the differentially expressed proteins obtained for the 46 targets, which all together have 169 SRM transitions, targeting the 63 peptides, are provided in S3 Table.

**Table 1:** Seven protein biomarker candidates specific for the LCNEC subtype identified by SRM-MS semi-quantitation and confirmed through statistical evaluations via both ANOVA and T-tests.

No.	Protein No. in SRM list	Protein entry name	Accession No.	Protein name	Peptide Sequence	SRM transitions:	Ave. SRM AUCs after the scaling by the reference			%-CV (*n* = 3)	ANOVA *p-value*	Pairwise *p-value* in T-test		
						[parent ion (*m/z*)/fragment ion (*m/z*)]	LCNEC^a^	LCC^b^	SCLC^c^			LCNEC vs LCC	LCNEC vs SCLC	LCC vs SCLC
1	2	4F2	P08195	4F2 cell surface antigen heavy chain	CQSEDPGSLLSLFR	753.4/1104.6	2661	514	412	88.8	1.20E-03	3.01E-03	3.17E-03	3.08E-01
						753.4/989.6	16984	3653	1215	50.2	3.00E-04	2.60E-03	1.06E-03	3.71E-03
2	4	AL1A1	P00352	Retinal dehydrogenase 1	IFVEESIYDEFVR	823.4/1385.7	7276	1241	566	27.8	1.00E-03	4.78E-03	2.48E-03	6.87E-02
						823.4/1286.6	7338	1139	586	48.5	7.00E-04	3.37E-03	2.26E-03	6.94E-02
						823.4/1157.5	6139	634	414	54.4	7.00E-04	2.77E-03	2.72E-03	2.46E-01
						823.4/1028.5	7147	1039	638	60.5	2.90E-03	1.10E-02	5.97E-03	1.67E-01
3	7	APOA1	P02647	Apolipoprotein A-I	VSFLSALEEYTK	693.9/1200.6	5691	945	1494	48.9	1.00E-04	4.19E-04	1.21E-03	1.55E-01
						693.9/1053.5	9314	2178	2296	56.8	1.00E-04	5.80E-04	6.80E-04	4.48E-01
						693.9/940.5	30383	7362	9014	65.4	1.00E-04	2.56E-04	7.68E-04	3.03E-01
						693.9/782.4	7937	1679	2389	21.1	1.00E-04	9.82E-05	7.61E-04	1.97E-01
					DYVSQFEGSALGK	700.8/1122.6	6240	2189	2029	58.1	1.00E-04	4.54E-04	3.87E-04	4.03E-01
						700.8/1023.5	28581	10383	9550	10.9	2.00E-04	7.25E-04	8.13E-04	3.84E-01
						700.8/808.4	18137	6836	6443	8.2	2.00E-04	3.93E-04	1.06E-03	4.22E-01
					LLDNWDSVTSTFSK	806.9/971.5	12419	1689	3488	52.3	1.00E-04	3.70E-04	2.22E-03	7.22E-02
						806.9/856.4	5034	979	1838	52.1	3.00E-04	7.85E-04	4.96E-03	5.24E-02
4	11	ENOB	P13929	Beta-enolase	VNQIGSVTESIQACK	788.9/1122.5	34708	14135	10241	10.8	2.60E-03	1.04E-02	3.23E-03	1.35E-01
						788.9/1065.5	4777	2223	1706	32.4	3.80E-03	9.72E-03	4.22E-03	1.75E-01
						788.9/879.4	14239	6479	4570	10.8	4.30E-03	1.62E-02	4.34E-03	8.36E-02
5	22	KCRB	P12277	Creatine kinase B-type	VLTPELYAELR	652.4/1091.6	43841	3774	19018	18.2	3.90E-03	3.34E-03	4.38E-02	1.84E-04
						652.4/990.5	130517	10191	59370	15.3	5.80E-03	3.52E-03	5.80E-02	9.25E-05
						652.4/893.5	29713	2582	11378	50.8	2.60E-03	1.25E-03	2.80E-02	4.71E-05
					DLFDPIIEDR	616.8/1004.5	32908	2264	16098	49.6	7.00E-03	2.44E-03	7.60E-02	8.52E-04
6	23	LG3BP	Q08380	Galectin-3-binding protein	ELSEALGQIFDSQR	796.9/950.5	21573	9302	3551	31.3	8.00E-03	3.59E-02	5.72E-03	3.73E-03
7	27	PEBP1	P30086	Phosphatidylethanolamine-binding protein 1	LYTLVLTDPDAPSR	780.9/971.5	26344	7038	16996	26.3	1.20E-03	3.30E-04	6.45E-02	5.45E-03
						780.9/858.4	19344	4605	11066	29.2	4.00E-04	3.79E-04	2.76E-02	2.32E-03

^a^LCNEC: Large-cell neuroendocrine lung carcinoma

^b^LCC: Large-cell lung cancer

^c^SCLC: Small-cell lung cancer

**Fig 4:**
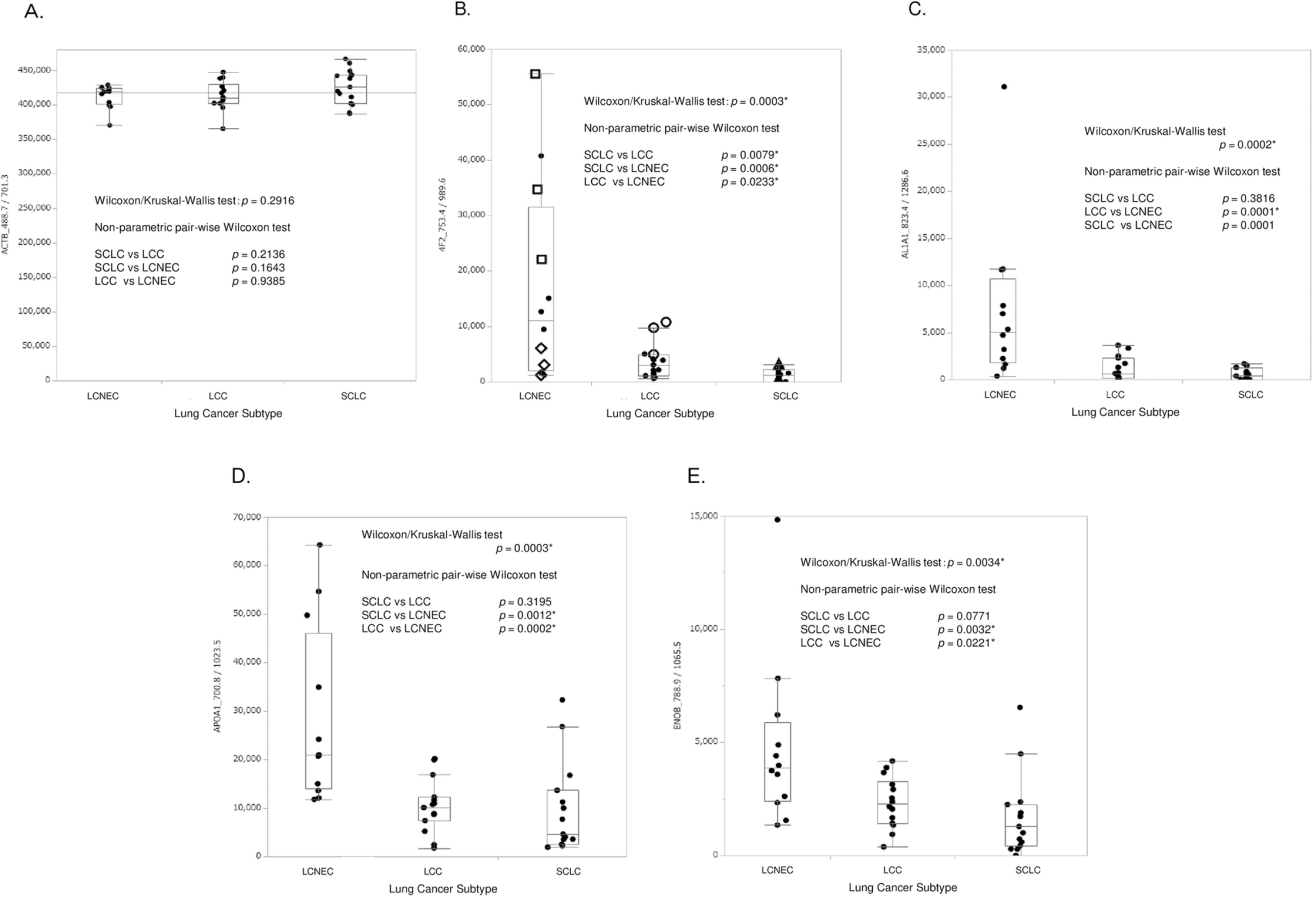
**Representative box plots of normalized SRM AUCs obtained for A) ACTB, B) 4F2hc, C) AL1A1, D) APOA1, and E) ENOB among the three lung cancer subtypes.** Data were produced in triplicate SRM-MS measurements and utilized after normalization by the endogenous protein reference. The normalized 4F2hc SRM AUCs correspond to the samples (□:LCNEC, Patient No. 1; ◇: LCNEC, Patient No. 4; ○: LCC, Patient No. 13; △: SCLC, Patient No. 24). The FFPE tissues subjected to SRM quantitation were obtained from LCNEC Patient No. 1–4, LCC Patient No. 11–15, and SCLC Patient No. 21–25. Results by both non-parametric Wilcoxon/Kruskal–Wallis and pairwise Wilcoxon tests are denoted in the figures.

A comparative analysis was made in-between the three lung cancer subtypes, and in Fig 4 these are illustrated with a series of representative box plots. These provide evidence of LCNEC separation from the other two subtypes, strengthening the validity of the protein candidates. The respective box plot is shown for the following protein candidates; A) ACTB, B) B4F2, C) AL1A1, D) APOA1, and E) ENOB, in which results include the statistical evaluations by both non-parametric statistical analysis, applying Wilcoxon/Kruskal–Wallis and pairwise Wilcoxon tests, since ANOVA *p*-values were affected by null values in some individual SRM AUCs. Fig 4A shows SRM AUCs obtained for the other SRM transition of ACTB: ACTB_488.7/701.3 [doubly-charged actin-β peptide AGFAGDDAPR (*m/z* 488.7) to the singly-charged *y*_*7*_ fragment (*m/z* 701.3)] measured in triplicate across all the 14 clinical samples. Fig 4B indicates the data of the normalized 4F2hc SRM AUCs corresponding to several samples (□:LCNEC, Patient No. 1; ◇: LCNEC, Patient No. 4; ○: LCC, Patient No. 13; △: SCLC, Patient No. 24).

For most of the resulting study outcomes, quantitative reproducibility was improved after applying the scaling method. However, some variations remain in normalized SRM AUCs for some of the patient samples, which can be attributed to technical variations in some LC/SRM- MS runs and/or in SRM AUC calculations from XICs. Technical improvement of LC/SRM-MS analysis would bring better quantitative results.

Those statistical evaluations showed that 4F2hc, AL1A1, ENOB and APOA1 are significant LCNEC biomarker candidates. Non-parametric pairwise Wilcoxon tests for KCRB, LG3BP, and PEBP1 suggested that both PEBP1 and KCRB would be relevant to NETs, and that LG3BP might be highly expressed in lung cancers of large-cell type, LCNEC and LCC. Thus, these proteins are not appropriate as the biomarker for LCNEC. The box plots of their respective SRM AUCs are provided in S1A–S1C Fig.

The 4F2hc protein is the heavy chain of CD98, a cell surface antigen [38]. The CD98, a type II transmembrane glycoprotein, consists of an 80–85 kD heavy chain (4F2hc/FRP-1) and a 40–45 kD light chain. Alterations in cell surface components often correlate with a tumorigenic cell phenotype. CD98 has multiple regulatory functions that include extracellular signaling, epithelial cell adhesion/polarity, amino acid transport, and cell-cell interaction [39].

**Fig 5:**
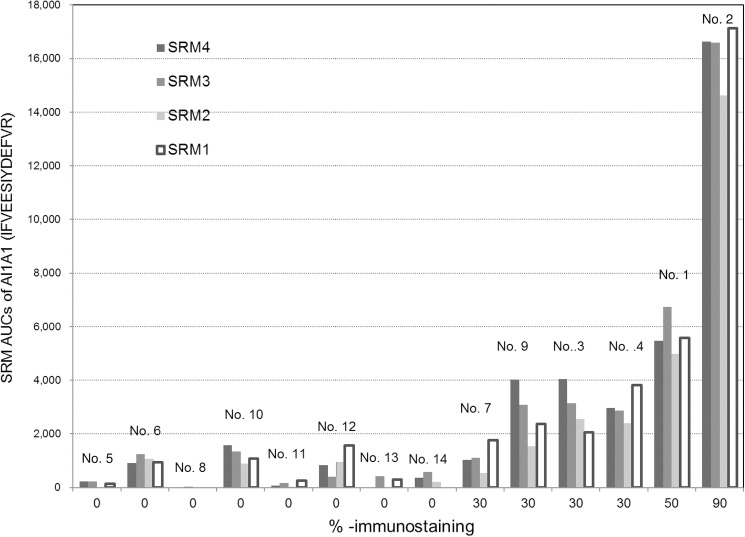
For each patient, the SRM AUCs are represented in histograms, together with patient numbers and %-values of immunostaining for AL1A1 (14 cases in S1 Table: LCNEC Patient No. 1–4, LCC Patient No. 11–15, SCLC Patient No. 21–25), in which the four SRM transitions for AL1A1 peptide, IFVEESIYDEFVR, [SRM1: *m/z* 823.4 (2+) → *m/z* 1028.5 (1+); SRM2: *m/z* 823.4 (2+) → *m/z* 1157.5 (1+); SRM3: *m/z* 823.4(2+) → *m/z* 1286.6 (1+); SRM4: *m/z* 823.4 (2+) → *m/z* 1385.7 (1+)] were used.

The AL1A1 candidate is not only a marker for stem cells, but may also play important functional roles related to self-protection, differentiation, and expansion [40–44]. For each patient, SRM-MS assay results (AUCs) of AL1A1 have been compared with antibody-based immunostaining measurements (as %-immunostaining). Data were obtained from FFPE tissue specimens of each patient. The SRM AUCs obtained for AL1A1 are depicted in the histogram in Fig 5, together with patient numbers and % -values of immunostaining for 14 cases: LCNEC Patient No. 1–4, LCC Patient No. 11–15, and SCLC Patient No. 21–25. The variation of normalized SRM AUCs for all the four SRM transitions along the patients seems to be reasonably consistent with that of %-immunostainings, although the two data sets cannot be directly compared. The %-immunostaining is the relative immuno-positive area (%) of cancerous lesions in a FFPE tissue on the slide. On the other hand, a SRM assay reflects the concentration of AL1A1 molecules (and/or its isoforms with and without posttranslational modifications) in the amount of tumor cells of interest collected by LCM.

APOA1, apolipoprotein A-I, is the major protein component of high-density lipoprotein (HDL). Whereas APOA1 is known as a highly abundant serum protein, APOA1 was recently reported to be expressed by lung cells [45]. In the preceding study [13], APOA1 showed elevated expression in LCNEC. However, APOA1 expression was not statistically significant, comparing LCNEC vs SCLC (*p* > 0.05 in G-test) but was statistically significant for both LCNEC vs LCC as well as SCLC vs LCC (*p* = 0.0032 and 0.0318 in G-test, respectively). The SRM-MS semi-quantitative results of APOA1, with three different peptide sequences, consistently confirmed its significant expression in LCNEC. It was, however, quite surprising that APOA1 was highly upregulated in the LCNEC subtype. The reason might be related to the emerging role of APOA1 in tumor biology and its potential role in cancer therapeutics due to its anti-tumorigenic effects mediated by multiple immunomodulatory mechanisms [46].

ENOB, was also found to be a biomarker candidate for the LCNEC subtype. γ-enolase (ENOG), also referred to as neuron specific enolase (NSE) [47], is a known NET marker along with secretagogin (SEGN) [48, 49]. In our study, 16 protein candidates were identified as potential NET markers, including SEGN, microtubule-associated protein 1B (MAP1B), small nuclear ribonucleoprotein G (RUXG), and transcription intermediary factor 1-β (TIF1B). We identified BASP1 as a novel marker candidate for SCLC and/or NET in this study. The box plots of SRM AUCs measured for ENOG, SEGN, and brain acid soluble protein 1 (BASP1) are provided in S1D–S1F Fig. It is known that BASP1 is a significant regulator of WT1 (the Wilms’ tumor suppressor) [50], and that BASP1 and WT1 cooperate to induce the differentiation of K562 cells to a neuronal-like morphology [51, 52], suggesting that BASP1 might be related to transcriptional reprogramming and morphological changes in lung cancer into a neuroendocrine phenotype, although its function in lung cancer remains unknown.

## Conclusions

The SRM-MS based semi-quantitation confirmed AL1A1 as a promising LCNEC biomarker candidate proposed in the preceding study [13], and also suggested that 4F2 (CD98), APOA1, and ENOB, would be useful in diagnosing the LCNEC subtype. The normalized SRM AUCs for AL1A1 showed a reasonable correspondence with their antibody-based %-IHCs. Though further technical development of this scaling method is needed to minimize experimental bias derived from protein expression measurements, this label-free semi-quantitative approach can already be used to select many highly promising biomarker candidates, particularly during the preliminary screening of the verification phase of biomarker development, in which a high number of protein candidates from the discovery phase need to be assessed.

LCNEC has a poor prognosis, similarly to SCLC, and the survival rate is only 18% in stage IA after resection. Currently resection is the first choice of treatment for this tumor type. Adjuvant chemotherapy is chosen for SCLC patients in most surgical cases. Multiple studies showed, taking into account the post-operative 5-year survival of patients, that the outcome of LCNEC was far worse than for other histological variants of NSCLC. Some studies revealed that the characteristics of LCNEC were more similar to SCLC than to LCC [53, 54]. There are no LCNEC biomarkers in clinical use today. Our data indicate that the combination of AL1A1 with a protein significant to SCLC, such as BASP1, might be useful to diagnose LCNEC, whereas NETs could be evaluated by expression of at least one of three representative neuroendocrine proteins: CD56, synaptophysin (Syn), or chromogranin A (CGA). Relative quantitative SRM- MS assays using the scaling method are feasible prior to further development processes involved in protein biomarkers, Based on the outcome of our study, we are confident that LCNEC will become easier to diagnose in the future as we found 7 LCNEC specific markers by evaluating 46 candidate proteins obtained from the comparative clinical proteomic study of three lung cancer subtypes: LCNEC, LCC, and SCLC [13].

## Supporting information

S1 Table Patients’ characteristics and immunoreactivity with biomarker candidates. Listed are patients’ characteristics and immunoreactivities with AL1A1, AK1C1, AK1C3, and CD44, and with antibodies raised against established neuroendocrine markers, CD56, CGA, and Syn. The immunoreactivity is indicated as the percentage of immunopositive area at the maximal cut-surface of tumors (Table data were taken and modified from the preceding study [13]).
(DOCX)

S2 Table The list of selected SRM transitions. The 169 SRM transitions for 63 peptides chosen to quantify targeted 46 proteins and their %-CV values (*n* = 4) in peak area (AUC) measured using the project control pooled sample.
(DOCX)

S3 Table The SRM-MS quantitation of targeted proteins. The full results obtained for the 46 proteins, including the 169 SRM transitions targeting the 63 peptides for LCNEC, LCC and SCLC (XLS).
(XLSX)

S1 Fig The box plots of SRM AUCs. A) KCRB, B) LG3BP, C) PEBP1, D) ENOG, E) SEGN, and F) BASP1, where also indicated are data corresponding to the following samples: □, LCNEC Patient No. 1; ◇, LCNEC Patient No.4; ○, LCC Patient No. 13; △, SCLC Patient No. 24, which are the same as denoted in Fig 3.
(TIF)
