# Supplementary material for: A selected reaction monitoring mass spectrometric assessment of biomarker candidates diagnosing large-cell neuroendocrine lung carcinoma by the scaling method using endogenous references
Source: PLoS One. 2017 Apr 27;12(4):e0176219. doi: 10.1371/journal.pone.0176219 (PMC5407814; doi:10.1371/journal.pone.0176219)
Supplement: S1 Table — Listed are patients’ characteristics and immunoreactivities with AL1A1, AK1C1, AK1C3, and CD44, and with antibodies raised against established neuroendocrine markers, CD56, CGA, and Syn. The immunoreactivity is indicated as the percentage of immunopositive area at the maximal cut-surface of tumors (Table data were taken and modified from the preceding study [13]). (DOCX) [file pone.0176219.s001.docx]

**S1 Table. Patients’ characteristics and immunoreactivity with biomarker candidates, AL1A1, AK1C1, AK1C3, and CD44, with antibodies raised against established neuroendocrine markers, CD56, CGA, and Syn. The immunoreactivity was indicated as the percentage of immunopositive area at the maximal cut-surface of tumors (Table data were taken and modified from the preceding study [13]).**

Ref [30]
