# Supplementary material for: A selected reaction monitoring mass spectrometric assessment of biomarker candidates diagnosing large-cell neuroendocrine lung carcinoma by the scaling method using endogenous references
Source: PLoS One. 2017 Apr 27;12(4):e0176219. doi: 10.1371/journal.pone.0176219 (PMC5407814; doi:10.1371/journal.pone.0176219)
Supplement: S2 Table — The 169 SRM transitions for 63 peptides chosen to quantify targeted 46 proteins and their %-CV values (n = 4) in peak area (AUC) measured using the project control pooled sample. (DOCX) [file pone.0176219.s002.docx]

**S2 Table. The 169 SRM transitions for 63 peptides chosen to quantify targeted 46 proteins and their %-CV values (*n*=4) in peak area (AUC) measured using the project control pooled sample, in which ‘-Homo sapiens (Human) ' was removed from protein names**

| No. | Protein Entry Name | SwissProt Accession No. | Protein Name | Peptide Sequense | SRM transitions: | Quantitative assessment: |
| --- | --- | --- | --- | --- | --- | --- |
|  |  |  |  |  | parent ion (*m/z)* / fragment ion (*m/z*) | %-CV peak area (*n*=4) |
|  |  |  |  |  |  |  |
| 1 | ACTB | P60709 | Actin, cytoplasmic 1 | AGFAGDDAPR | 488.7 / 701.3 | 12.0 |
|  |  |  |  |  | 488.7 / 630.3 | 8.0 |
| 2 | 4F2 | P08195 | 4F2 cell-surface antigen heavy chain | CQSEDPGSLLSLFR | 753.4 / 989.6 | 50.2 |
|  |  |  |  |  | 753.4 / 1104.6 | 88.8 |
| 3 | AHNK | Q09666 | Neuroblast differentiation-associated protein AHNAK | GEGPDVDVNLPK | 620.3 / 784.5 | 51.3 |
|  |  |  |  |  | 620.3 / 685.4 | 50.8 |
|  |  |  |  | AEGPEVDVNLPK | 634.3 / 685.4 | 45.6 |
|  |  |  |  |  | 634.3 / 1067.6 | 49.6 |
| 4 | AL1A1 | P00352 | Retinal dehydrogenase 1 | IFVEESIYDEFVR | 823.4 / 1385.7 | 27.8 |
|  |  |  |  |  | 823.4 / 1286.6 | 48.5 |
|  |  |  |  |  | 823.4 / 1157.5 | 54.4 |
|  |  |  |  |  | 823.4 / 1028.5 | 60.5 |
| 5 | ANXA2 | P07355 | Annexin A2 | TPAQYDASELK | 611.8 / 953.5 | 26.3 |
|  |  |  |  |  | 611.8 / 825.4 | 25.5 |
|  |  |  |  |  | 611.8 / 662.3 | 10.1 |
|  |  |  |  |  | 611.8 / 1024.5 | 44.4 |
| 6 | ANXA6 | P08133 | Annexin A6 | ALIEILATR | 500.3 / 815.5 | 29.1 |
|  |  |  |  |  | 500.3 / 702.4 | 37.1 |
|  |  |  |  |  | 500.3 / 573.4 | 29.7 |
| 7 | APOA1 | P02647 | Apolipoprotein A-I precursor | DYVSQFEGSALGK | 700.8 / 808.4 | 8.2 |
|  |  |  |  |  | 700.8 / 1122.6 | 58.1 |
|  |  |  |  |  | 700.8 / 1023.5 | 10.9 |
|  |  |  |  | LLDNWDSVTSTFSK | 806.9 / 971.5 | 52.3 |
|  |  |  |  |  | 806.9 / 856.4 | 52.1 |
|  |  |  |  | VSFLSALEEYTK | 693.9 / 940.5 | 65.4 |
|  |  |  |  |  | 693.9 / 782.4 | 21.1 |
|  |  |  |  |  | 693.9 / 1200.6 | 48.9 |
|  |  |  |  |  | 693.9 / 1053.5 | 56.8 |
| 8 | BASP1 | P80723 | Brain acid soluble protein 1 | ETPAATEAPSSTPK | 693.8 / 988.5 | 13.5 |
|  |  |  |  |  | 693.8 / 917.5 | 21.9 |
|  |  |  |  |  | 693.8 / 816.4 | 32.1 |
| 9 | CALU | O43852 | Calumenin precursor | TFDQLTPEESK | 647.8 / 690.3 | 33.6 |
|  |  |  |  |  | 647.8 / 1046.5 | 9.4 |
| 10 | CATD | P07339 | Cathepsin D precursor | VGFAEAAR | 410.7 / 721.4 | 8.6 |
|  |  |  |  |  | 410.7 / 664.3 | 21.0 |
|  |  |  |  |  | 410.7 / 517.3 | 10.6 |
|  |  |  |  |  | 410.7 / 446.2 | 5.6 |
|  |  |  |  | YYTVFDR | 482.2 / 637.3 | 28.2 |
|  |  |  |  |  | 482.2 / 536.3 | 22.4 |
|  |  |  |  | FDGILGMAYPR | 620.3 / 807.4 | 54.8 |
|  |  |  |  |  | 620.3 / 694.3 | 33.1 |
| 11 | ENOB | P13929 | Beta-enolase | VNQIGSVTESIQACK | 788.9 / 879.4 | 10.8 |
|  |  |  |  |  | 788.9 / 1235.6 | 44.6 |
|  |  |  |  |  | 788.9 / 1122.5 | 10.8 |
|  |  |  |  |  | 788.9 / 1065.5 | 32.4 |
| 12 | ENOG | P13929 | Gamma-enolase | IEEELGDEAR | 580.8 / 918.4 | 18.2 |
|  |  |  |  |  | 580.8 / 789.4 | 15.6 |
|  |  |  |  |  | 580.8 / 660.3 | 13.8 |
| 13 | FABP5 | Q01469 | Fatty acid-binding protein, epidermal | FEETTADGR | 513.2 / 749.3 | 14.5 |
|  |  |  |  |  | 513.2 / 620.3 | 20.1 |
| 14 | FABP7 | O15540 | Fatty acid-binding protein, brain | NTEISFQLGEEFDETTADDR | 1159.0 / 1497.6 | 115.5 |
|  |  |  |  |  | 1159.0 / 1384.5 | 86.1 |
| 15 | FAS | P49327 | Fatty acid synthase | LQVVDQPLPVR | 632.4 / 923.5 | 36.0 |
|  |  |  |  |  | 632.4 / 1022.6 | 24.8 |
| 16 | FINC | P02751 | Fibronectin precursor | VPGTSTSATLTGLTR | 731.4 / 919.5 | 22.2 |
|  |  |  |  |  | 731.4 / 832.5 | 23.9 |
|  |  |  |  |  | 731.4 / 1107.6 | 11.7 |
|  |  |  |  |  | 731.4 / 1020.6 | 19.0 |
|  |  |  |  | SYTITGLQPGTDYK | 772.4 / 978.5 | 12.9 |
|  |  |  |  |  | 772.4 / 1079.5 | 22.9 |
| 17 | FSCN1 | Q16658 | Fascin | FLIVAHDDGR | 571.8 / 769.4 | 32.9 |
|  |  |  |  |  | 571.8 / 670.3 | 57.5 |
|  |  |  |  |  | 571.8 / 599.3 | 47.2 |
| 18 | GELS | P06396 | Gelsolin precursor | TGAQELLR | 444.2 / 786.4 | 28.7 |
|  |  |  |  |  | 444.2 / 729.4 | 22.3 |
|  |  |  |  |  | 444.2 / 658.4 | 25.3 |
|  |  |  |  |  | 444.2 / 530.3 | 20.7 |
| 19 | HSPB1 | P04792 | Heat shock protein beta-1 | AQLGGPEAAK | 471.3 / 742.4 | 15.3 |
|  |  |  |  |  | 471.3 / 629.3 | 13.3 |
|  |  |  |  |  | 471.3 / 572.3 | 5.5 |
|  |  |  |  | LATQSNEITIPVTFESR | 953.5 / 835.4 | 21.5 |
|  |  |  |  |  | 953.5 / 1049.6 | 9.8 |
|  |  |  |  | LFDQAFGLPR | 582.3 / 903.5 | 26.5 |
|  |  |  |  |  | 582.3 / 788.4 | 21.6 |
|  |  |  |  |  | 582.3 / 660.4 | 26.4 |
| 20 | IDHP | P48735 | Isocitrate dehydrogenase [NADP], mitochondrial | TIEAEAAHGTVTR | 678.3 / 941.5 | 52.2 |
|  |  |  |  |  | 678.3 / 741.4 | 25.4 |
| 21 | IMB1 | Q14974 | Importin subunit beta-1 | VLANPGNSQVAR | 613.3 / 942.5 | 21.7 |
|  |  |  |  |  | 613.3 / 828.4 | 8.1 |
|  |  |  |  |  | 613.3 / 1013.5 | 18.2 |
|  |  |  |  | AAVENLPTFLVELSR | 830.0 / 1417.8 | 62.4 |
|  |  |  |  |  | 830.0 / 1174.7 | 45.8 |
|  |  |  |  |  | 830.0 / 1061.6 | 39.4 |
| 22 | KCRB | P12277 | Creatine kinase B-type | VLTPELYAELR | 652.4 / 990.5 | 15.3 |
|  |  |  |  |  | 652.4 / 893.5 | 50.8 |
|  |  |  |  |  | 652.4 / 1091.6 | 18.2 |
|  |  |  |  | DLFDPIIEDR | 616.8 / 857.4 | 31.3 |
|  |  |  |  |  | 616.8 / 742.4 | 27.2 |
|  |  |  |  |  | 616.8 / 1004.5 | 49.6 |
| 23 | LG3BP | Q08380 | Galectin-3-binding protein precursor | ELSEALGQIFDSQR | 796.9 / 950.5 | 31.3 |
|  |  |  |  |  | 796.9 / 1350.7 | 81.3 |
| 24 | MAP1B | P46821 | Microtubule-associated protein 1B | AIGNIELGIR | 528.3 / 871.5 | 13.0 |
|  |  |  |  |  | 528.3 / 587.4 | 24.5 |
| 25 | MVP | Q14764 | Major vault protein | LAQDPFPLYPGEVLEK | 908.5 / 1388.7 | 11.7 |
|  |  |  |  |  | 908.5 / 1144.6 | 45.2 |
|  |  |  |  | TAVFGFETSEAK | 643.8 / 868.4 | 4.1 |
|  |  |  |  |  | 643.8 / 811.4 | 25.8 |
|  |  |  |  |  | 643.8 / 664.3 | 32.4 |
|  |  |  |  |  | 643.8 / 1015.5 | 26.6 |
| 26 | NAPSA | O96009 | Napsin-A precursor | FAIQYGTGR | 506.8 / 681.3 | 35.4 |
|  |  |  |  |  | 506.8 / 553.3 | 48.3 |
| 27 | PEBP1 | P30086 | Phosphatidylethanolamine-binding protein 1 | LYTLVLTDPDAPSR | 780.9 / 971.5 | 26.3 |
|  |  |  |  |  | 780.9 / 858.4 | 29.2 |
| 28 | PGS1 | P21810 | Biglycan | IQAIELEDLLR | 656.9 / 887.5 | 15.5 |
|  |  |  |  |  | 656.9 / 1071.6 | 78.4 |
|  |  |  |  |  | 656.9 / 1000.6 | 44.5 |
| 29 | PLEC1 | Q15149 | Plectin-1 | SQVEEELFSVR | 661.8 / 879.5 | 37.3 |
|  |  |  |  |  | 661.8 / 1107.6 | 30.9 |
|  |  |  |  |  | 661.8 / 1008.5 | 16.3 |
| 30 | PLSL | P13796 | Plastin-2 | NEALIALLR | 506.8 / 769.5 | 28.1 |
|  |  |  |  |  | 506.8 / 698.5 | 31.3 |
|  |  |  |  |  | 506.8 / 585.4 | 44.0 |
|  |  |  |  | EITENLMATGDLDQDGR | 939.4 / 1178.5 | 57.4 |
|  |  |  |  |  | 939.4 / 1047.5 | 51.0 |
| 31 | POSTIN | Q15063 | Periostin precursor | AAAITSDILEALGR | 700.9 / 771.5 | 34.9 |
|  |  |  |  |  | 700.9 / 1074.6 | 52.0 |
| 32 | PROF1 | P07737 | Profilin-1 | TFVNITPAEVGVLVGK | 822.5 / 968.6 | 33.4 |
|  |  |  |  |  | 822.5 / 1069.6 | 41.9 |
| 33 | PSPB | P0798 | Pulmonary surfactant-associated protein B precursor | YSVILLDTLLGR | 681.9 / 900.6 | 29.6 |
|  |  |  |  |  | 681.9 / 787.5 | 77.8 |
| 34 | RCN1 | Q15293 | Reticulocalbin-1 precursor | TFDQLTPDESK | 640.8 / 789.4 | 26.2 |
|  |  |  |  |  | 640.8 / 676.3 | 25.8 |
|  |  |  |  | EIVVLETLEDIDK | 758.4 / 962.5 | 59.8 |
|  |  |  |  |  | 758.4 / 833.4 | 80.2 |
|  |  |  |  |  | 758.4 / 1174.6 | 51.8 |
|  |  |  |  |  | 758.4 / 1075.6 | 46.8 |
| 35 | RUXG | P62308 | Small nuclear ribonucleoprotein G | HVQGILR | 411.7 / 685.4 | 29.0 |
|  |  |  |  |  | 411.7 / 586.4 | 25.8 |
|  |  |  |  |  | 411.7 / 458.3 | 28.7 |
| 36 | S10A4 | P26447 | Protein S100-A4 | TDEAAFQK | 455.2 / 808.4 | 32.2 |
|  |  |  |  |  | 455.2 / 693.4 | 18.1 |
|  |  |  |  |  | 455.2 / 564.3 | 12.3 |
| 37 | S10A9 | P06702 | Protein S100-A9 | LGHPDTLNQGEFK | 728.4 / 799.4 | 115.5 |
|  |  |  |  |  | 728.4 / 1148.6 | 45.1 |
|  |  |  |  |  | 728.4 / 1051.5 | 54.7 |
| 38 | SAMP | P02743 | Serum amyloid P-component precursor | IVLGQEQDSYGGK | 697.3 / 1181.5 | 45.2 |
|  |  |  |  |  | 697.3 / 1068.5 | 22.6 |
|  |  |  |  | VGEYSLYIGR | 578.8 / 871.5 | 37.1 |
|  |  |  |  |  | 578.8 / 708.4 | 31.5 |
|  |  |  |  |  | 578.8 / 621.4 | 38.9 |
| 39 | SEGN | O76038 | Secretagogin | LDAAGFWQVWQR | 738.9 / 902.5 | 14.1 |
|  |  |  |  |  | 738.9 / 1106.6 | 78.4 |
| 40 | SERPH | P50454 | Serpin H1 precursor | DEEVHAGLGELLR | 719.4 / 965.6 | 37.2 |
|  |  |  |  |  | 719.4 / 828.5 | 22.9 |
|  |  |  |  |  | 719.4 / 757.5 | 39.6 |
|  |  |  |  | DTQSGSLLFIGR | 647.3 / 862.5 | 34.0 |
|  |  |  |  |  | 647.3 / 718.5 | 30.0 |
| 41 | SODC | P00441 | Superoxide dismutase [Cu-Zn] | HVGDLGNVTADK | 613.3 / 989.5 | 9.8 |
|  |  |  |  |  | 613.3 / 817.4 | 9.6 |
|  |  |  |  |  | 613.3 / 1088.6 | 15.4 |
| 42 | STMN1 | P16949 | Stathmin | AIEENNNFSK | 583.3 / 981.4 | 25.3 |
|  |  |  |  |  | 583.3 / 852.4 | 37.2 |
|  |  |  |  |  | 583.3 / 723.3 | 19.8 |
|  |  |  |  | ASGQAFELILSPR | 694.9 / 974.6 | 20.6 |
|  |  |  |  |  | 694.9 / 827.5 | 22.1 |
|  |  |  |  |  | 694.9 / 1045.6 | 24.0 |
| 43 | TAGL | Q01995 | Transgelin | EFTESQLQEGK | 648.3 / 789.4 | 33.9 |
|  |  |  |  |  | 648.3 / 1019.5 | 16.7 |
| 44 | TIF1B | Q13263 | Transcription intermediary factor 1-beta | LDLDLTADSQPPVFK | 829.9 / 988.5 | 17.5 |
|  |  |  |  |  | 829.9 / 1202.6 | 28.3 |
|  |  |  |  |  | 829.9 / 1089.6 | 32.0 |
| 45 | TIMP3 | P35625 | Metalloproteinase inhibitor 3 precursor | WDQLTLSQR | 573.8 / 960.5 | 22.3 |
|  |  |  |  |  | 573.8 / 717.4 | 20.2 |
| 46 | VIME | P08670 | Vimentin | VESLQEEIAFLK | 703.4 / 977.5 | 41.2 |
|  |  |  |  |  | 703.4 / 1177.6 | 28.9 |
|  |  |  |  |  | 703.4 / 1090.6 | 16.1 |
|  |  |  |  | ILLAELEQLK | 585.4 / 943.5 | 32.2 |
|  |  |  |  |  | 585.4 / 830.5 | 22.6 |
|  |  |  |  |  | 585.4 / 759.4 | 23.8 |
|  |  |  |  |  |  |  |
|  |  |  |  |  |  |  |
