# Supplementary figures and images for: A selected reaction monitoring mass spectrometric assessment of biomarker candidates diagnosing large-cell neuroendocrine lung carcinoma by the scaling method using endogenous references
Source: PLoS One. 2017 Apr 27;12(4):e0176219. doi: 10.1371/journal.pone.0176219 (PMC5407814; doi:10.1371/journal.pone.0176219)

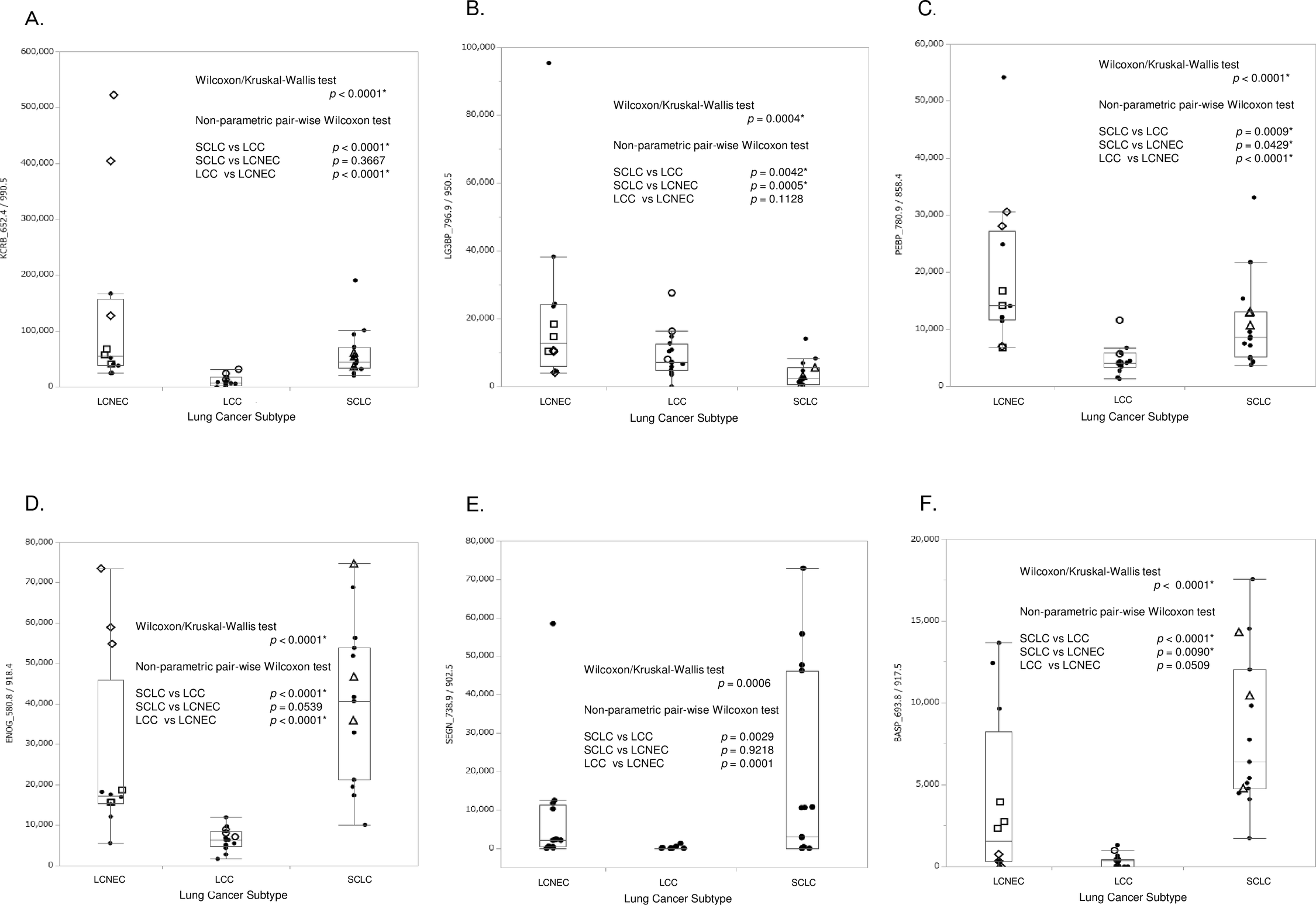

Supplement: S1 Fig — A) KCRB, B) LG3BP, C) PEBP1, D) ENOG, E) SEGN, and F) BASP1, where also indicated are data corresponding to the following samples: □, LCNEC Patient No. 1; ◇, LCNEC Patient No.4; ○, LCC Patient No. 13; △, SCLC Patient No. 24, which are the same as denoted in Fig 3. (TIF) [file pone.0176219.s004.tif]
